# Comprehensive evaluation of effects and safety of statin on the progression of liver cirrhosis: a systematic review and meta-analysis

**DOI:** 10.1186/s12876-019-1147-1

**Published:** 2019-12-30

**Authors:** Yue Gu, Xueqin Yang, Hang Liang, Deli Li

**Affiliations:** 1 Department of Hepatobiliary and Pancreatic Surgery, First Hospital of Jilin University, Changchun, Jilin China; 2 Department of Traditional Chinese Medicine, First Hospital of Jilin University, Changchun, Jilin China; 3 Department of Pediatric Respiratory II, First Hospital of Jilin University, No. 1 Xinmin Street, Changchun, 130021 Jilin China

**Keywords:** Statin, Liver cirrhosis, Portal hypertension, Complication, Meta-analysis

## Abstract

**Background:**

Statin has been more and more widely used in chronic liver disease, however, existed studies have attained contradictory results. According to the present study, we aimed to test the efficacy and safety of statin via a meta-analysis.

**Methods:**

Different databases were searched for full-text publication based on inclusion and exclusion criteria. For data-pooling, fixed-effect model was applied if heterogeneity wasn’t detected. Otherwise, random-effect model was adopted. Heterogeneity was detected by I squire (*I*^*2*^) test. All results of analysis were illustrated as forest plots. Publication bias was assessed using the Begg’s adjusted rank correlation test. Standard mean difference (SMD) was calculated in continuous variables. Pooled hazard ratio or odds ratio was calculated in catergorical variables.

**Results:**

Seventeen clinical studies were finally included. Hepatic portal hemodynamic parameters were improved in statin users for a short-term response. For a long-term follow-up, statin treatment surprisingly decreased mortality rate (HR = 0.782, 95% CI: 0.718–0.846, *I*^*2*^ > 50%) and lower the occurrence of hepatocellular carcinoma (HR = 0.75, 95% CI: 0.64–0.86, *I*^*2*^ > 50%) in liver cirrhosis. Statin seemed not to decrease the risk of esophageal variceal bleeding and spontaneous bacterial peritonitis. However, statin was proved to decrease the risk of hepatic encephalopathy and ascites. Incidence of drug related adverse events didn’t increase in statin users. Dose-dependent effects of statin on hepatocellular carcinoma development, decompensated cirrhosis events occurrence, and liver cirrhosis progression.

**Conclusion:**

Statin influenced parameters of hepatic portal vessel pressure in short-term treatment. Prognosis of liver cirrhosis benefited from statin treatment in long term follow-up. The efficacy and safety of statin in liver cirrhosis treatment is confirmed. To date, similar study is hardly seen before.

## Background

The major causes of liver cirrhosis are: alcoholic liver disease (ALD), chronic viral hepatitis (chronic hepatitis B, chronic hepatitis C), non-alcoholic fatty liver disease (NAFLD) or non-alcoholic steatohepatitis (NASH), and others such as primary biliary cholangitis (primary biliary cirrhosis, PBC), primary sclerosing cholangitis (PSC), autoimmune hepatitis, hemochromatosis, Wilson’s disease, alpha-metabolic diseases such as: 1-antitrypsin deficiency, galactosemia and glycogen storage disorders, and heart failure with liver congestion [1]. After liver cirrhosis developed into decompensated cirrhosis, mortality rate would astoundingly increase [2]. Nowadays, liver cirrhosis has become one of the most deadly disease all over the world [3, 4], and hepatic encephalopathy, variceal haemorrhage, spontaneous bacterial peritonitis, and hepatocellular carcinoma (HCC), etc. are listed as the main cause of death in liver cirrhosis [5]. The disease progression could be hardly reversed when decompensated liver cirrhosis is developed, and therefore, early intervention of preventive medication may play an important role to fight against liver cirrhosis and improve its prognosis.

Statins is a set of lipid-lowering agents by targeting at inhibiting the activity of 3-hydroxy-3-methylglutaryl co-enzyme A (HMG-CoA) reductase, resulting in inhibition of cholesterol generation and serum cholesterol levels downregulation [6, 7]. Except for its well-acknowledged function, decreasing serum low density lipoprotein C cholesterol, statin is also believed to alleviate oxidative stress injury, prohibit inflammatory cell activation, reduce the level of inflammation reaction, and improve endothelial function through a nitric oxide synthase dependent pathway [8–11]. Recent years, statin has been more and more widely used in chronic liver disease [12, 13], and it draws a lot of interests in investigating the good effects of statins on the primary prevention and secondary prevention of liver cirrhosis. Retrospective cohort studies in large populations of patients with cirrhosis and pre-cirrhotic conditions have shown that treatment with statins, with the purpose of decreasing high cholesterol levels, was associated with a reduced risk of disease progression, hepatic decompensation, hepatocellular carcinoma development, and death. Finally, a few randomised controlled trials (RCTs) have shown that treatment with simvastatin decreases portal pressure (two studies) and mortality (one study). Statin treatment was generally well tolerated but a few patients developed severe side effects, particularly rhabdomyolysis. Despite these promising beneficial effects, further RCTs are required, with larger patient series and hard clinical endpoints should be performed before statins can be recommended for use in patients with chronic liver disease [14–19]. However, statins itself could lead to hepatic dysfunction [6], especially in combination with the drug which is metabolised by cytochrome P450 enzyme system [20]. Considering the potential hepatotoxicity of statins, its benefits in liver cirrhosis might be dampened. Besides, existed studies concerning statins treatments in preventing liver cirrhosis have attained contradictory results somehow [21–37]. Consequently, a systematic study to synthesize data from different studies to test the efficacy and safety of statin in liver cirrhosis treatment is highly needed. To date, similar study is hardly seen before, so that we aim to comprehensively evaluate the statin on liver cirrhosis and its development.

## Methods

### Search strategy

This study design was stringently conformed to the Preferred Reporting Items for Systematic Reviews and Meta-Analyses (PRISMA) statement [38]. Five databases, namely Pubmed, MEDLINE on Ovid, EBSCO, Web of Science, and the Cochrane Library, were searched as mentioned before with key words such as statin, liver cirrhosis, hepatic portal hypertension, decompensated cirrhosis, and complication to retrieve related literature published before June 2019.

### Study selection criteria and data extraction

Two investigators who were not informed with the protocol of the present study checked the quality and eligibility of all retrieved studies and collected the data independently. The finally included literature met criteria as follows: English language; comparison between statin treatment group and non-statin treatment group; with full-text instead of abstract only; clear definition of decompensation events of liver cirrhosis and statin related complications, e.g. variceal haemorrhage, ascites, hepatic encephalopathy, diarrhea, and myalgia, etc. Exclusion criteria included: animal studies (basic research); case-reports, case-series, and reviews articles; acute hemodynamic study. In cases of different publications from the same study, the one with the most complete data was chosen. Interested data such as the number of total patients and the number of patients with clearly defined events were carefully collected. Besides, basic demographic data and follow-up duration were collected as well [39–42].

### Data synthesis and statistical analysis

If non-heterogeneity was detected, fixed-effects models were introduced to integrate data to compare statin treatment or not in the difference of short term follow-up and long term follow-up. Other than, the random-effects models were adopted. Heterogeneity was detected by I squire (*I*^*2*^) test. Heterogeneity was defined as *I*^*2*^ < 50%, and the value of *I*^*2*^ was shown in the forest plot. Results were presented as pooled hazard ratio (HR) or pooled odds ratio (OR). All results of analysis were illustrated as forest plots to make them visualizable. Additionally, publication bias was assessed using the Begg’s adjusted rank correlation test and shown as funnel plot [39–44], and Additional file 1: Figure S1 showed the typical diagram of publication bias analysis. The Newcastle-Ottawa Scale was used to evaluate the quality of each study independently, and quality assessment results were presented in Table 1.

**Table 1:** Quality assement of eligible literatures

Quality Assesment								
RCT study	Author	Prospective design	Clear definition of study population	(1)	(2)	(3)	(4)	(5)
	Abraldes, et al. 2009 [21]	Yes	Yes	Yes	Stable	Yes	Yes	Yes
	Pollo-Flores, et al. 2015 [32]	Yes	Yes	Yes	Stable	Yes	Yes	Yes
	Abraldes, et al. 2016 [22]	Yes	Yes	Yes	Stable	Yes	Yes	Yes
	Bishnu, et al. 2018 [24]	Yes	Yes	Yes	Stable	Yes	Yes	Yes
	Elwan, et al. 2018 [26]	Yes	Yes	Yes	Stable	Yes	Yes	Yes
	Domenico, et al	Yes	Yes	Yes	Stable	Yes	Yes	Yes
non-RCT study	Author	Study design	Clear definition of study population	Clear definition of different type of statin	Clear definition of related endpoints	Blindness to a statin or placebo	Representativeness of the study population	Comparability between case and control groups
	Kumar, et al. 2014 [30]	Propensity Score Matching Case-control Study	Yes	Yes	Yes	No	Yes	Yes
	Simon, et al. 2015 [34]	Cohort study	Yes	No	Yes	Not given	Yes	Yes
	Yang, et al. 2015 [37]	Propensity Score Matching Case-control Study	Yes	No	Yes	No	Yes	Yes
	Huang, et al. 2017 [27]	Propensity Score Matching Case-control Study	Yes	No	Yes	No	Yes	Yes
	Mohanty, et al. 2016 [31]	Propensity Score Matching Case-control Study	Yes	Yes	Yes	Not given	Yes	Yes
	Simon, et al. 2016 [33]	Case-control Study	Yes	Yes	Yes	No	Yes	Yes
	Bang, et al. 2017 [23]	Case-control Study	Yes	No	Yes	No	Yes	Yes
	Chang, et al. 2017 [25]	Propensity Score Matching Case-control Study	Yes	No	Yes	Not given	Yes	Yes
	Kim, et al. 2017 [13]	Nested Case-control Study	Yes	Yes	Yes	No	Yes	Yes
	Wong, et al. 2017 [36]	Propensity Score Matching Case-control Study	Yes	Yes	Yes	No	Yes	Yes
	Wani, et al. 2017 [35]	Self-control Study (Prospective Cohort)	Yes	Yes	Yes	No	Yes	Yes
	Kaplan, et al. 2019 [28]	Propensity Score Matching Case-control Study	Yes	No	Yes	Not given	Yes	Yes

(1) whether the study design was suitable for the disease condition and statin treatment; (2) were statin treatment stable or fluctuating; (3) was different cohort comparable to each other; (4) was there any clear definition of end event; (5) was plan of follow-up clearly given

### Statistics

Statistical heterogeneity was measured using the Inverse Variance (I-V) statistics. Statistical analyses were performed using Stata software 12.0 (Stata Corp, College Station, Texas). Standard mean difference (SMD) was calculated in continuous variables, and pooled HR value or pooled OR value was calculated in catergorical variables. All *p* values were 2-tailed, and the statistical significance was set at 0.05 (95% confidence interval).

## Results

### Demography of included studies at baseline

**Fig. 1:**
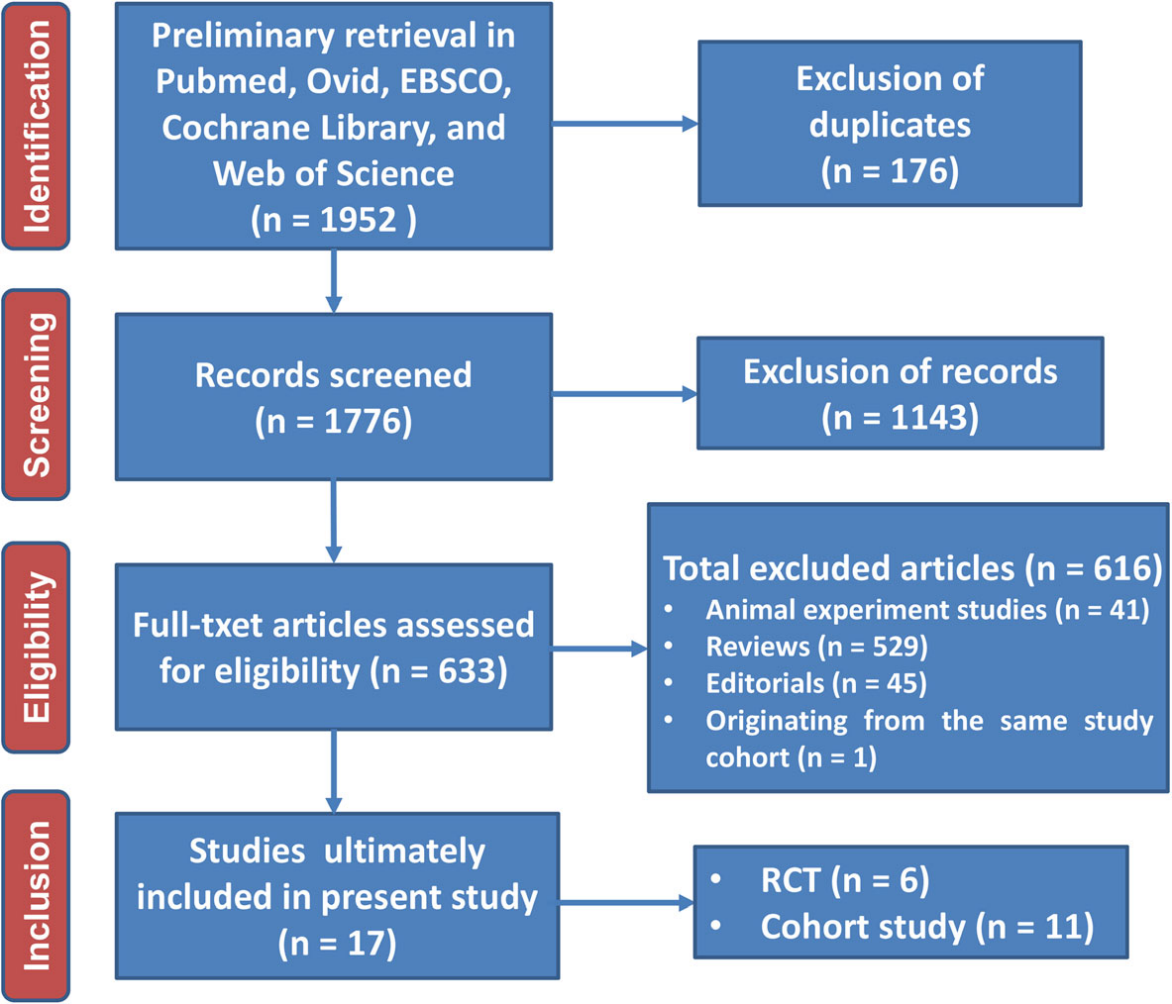
Flow diagram of literature filtration

Literature search, data extraction, and general description of included studies were carried out by two independent researchers. Total of 1776 articles was searched after excluding 176 duplications. Six hundred sixteen articles were excluded afterwards for not meeting the inclusion/exclusion criteria. Based on the aim of the present study, 17 clinical studies were finally included [21–37]. The flow diagram of publication filtration was shown in Fig. 1. Demographic data of patients with short term follow-up and long term follow-up were pooled together, respectively. The characteristics of included studies were generally described in Tables 2 and 3.

**Table 2:** Demography of patients in included studies

Author	Year	Age (year)		Male (%)		Number		Aetiology of liver disease									
								Alcoholic		HBV		HCV		NAFLD		Others	
		Control	Statin	Control	Statin	Control	Statin	Control	Statin	Control	Statin	Control	Statin	Control	Statin	Control	Statin
Abraldes, et al	2009	56 ± 10	58 ± 10	21	17	27	28	12	11	2	0	13	4	–	–	0	3
Kumar, et al	2014	59.6 ± 10.6	59.8 ± 10.9	88 (54.32)	44 (54.3)	162	81	39 (24.1)	18 (22.2)	10 (6.2)	2 (2.5)	55 (34)	18 (22.2)	41 (25.3)	35 (43.2)	6 (3.7)	3 (3.7)
Pollo-Flores, et al	2015	58.5 ± 13.5	56.5 ± 8.7	50%	57%	20	14	4	3	5	4	7	9	none	none	1	1
Simon, et al	2015	50.1 ± 7.2	54.2 ± 7.2	71.2	58.6	514	29	none	none	none	none	all	all	none	none	none	none
Yang, et al	2015	NG	NG	23,602 (42)	11,801 (42)	56,142	28,071	none	none	none	none	all	all	none	none	none	none
Mohanty, et al	2016	54 (50–58)	56 (52–60)	671 (97.9)	677 (98.8)	685	685	none	none	none	none	all	all	none	none	none	None
Huang, et al	2016	49.7 ± 11.5	50 ± 11.1	3479 (53.2)	3454 (52.8)	6543	6543	none	none	all	all	none	none	none	none	none	None
Simon, et al	2016	52.5 ± 6.9	53.5 ± 5.9	95.37	96.16	4970	4165	none	none	none	none	all	all	none	none	none	none
Abraldes, et al	2016	57.6 ± 10.6	57.4 ± 11.3	53 (67.9)	45 (65.2)	78	69	55 (71.4)	49 (71)	2 (2.6)	1 (1.4)	17 (22.1)	19 (27.5)	4 (5.2)	1 (1.4)	11	6
Wani, et al	2017	58.5 ± 6	58.5 ± 6	21	21	38	38	12	12	15	15	15	15	11	11	none	none
Wong, et al	2017	59.9 ± 13.9	60 ± 13.1	21,835 (58.8)	1266 (61.7)	67,131	2053	none	none	61,692 (89.7)	1867 (90.9)	4925 (8.9)	158 (7.7)	none	none	514 (0.8)	28 (1.4)
Bang, et al	2017	54 ± 10	57 ± 9	60%	61%	496	248	all	all	none	none	none	none	none	none	none	None
Chang, et al	2017	57.5 ± 14.1	56.5 ± 11.2	476 (71)	492 (73)	675	675	231 (34)	216 (32)	292 (43)	313 (46)	152 (23)	146 (22)	none	none	none	none
Kim, et al	2017	61.8 ± 9.2	61.8 ± 9.2	6860 (83.6)	1372 (83.6)	8210	1642	not given									
Bishnu, et al	2018	46.7 (7.1)	44 ± 12.7	12 (100)	9 (81.2)	12	11	6 (50)	4 (36.4)	1 (8.3)	0	0	0	1 (8.33)	0	1 (8.33)	1 (9.09)
Kaplan, et al	2019	63 (58–68)	63 (58–67)	98%	98	12,860	6481	4876 (35.2)	2334 (36)	none	none	2065 (14.9)	933 (14.4)	2159 (15.6)	1042 (16.1)	none	none
Elwan, et al.	2019	50.8 ± 7	51.5 ± 6.7	16 (80)	10 (50)	20	20	none	none	HCV 38, HBV 1, HCV + HBV 1					none	none	none

*SD* Standard deviation, *IQR* Interquartile range, *HBV* Hepatitis B virus, *HCV* Hepatitis C virus, *NAFLD* Non-alcoholic fatty liver disease

**Table 3:** Characteristics of included studies

Author	Year	Number of patients	Study design	Statin	Follow-up duration
Abraldes, et al	2009	55	Randomized controlled trial	simvastatin	1 month
Kumar, et al	2014	243	Propensity Score Matching Case-control Study	atorvastatin, fluvastatin, lovastatin, pravastatin, rosuvastatin and simvastatin	13 years
Simon, et al	2015	543	Cohort study	non-selected	3.5 years
Yang, et al	2015	84,213	Propensity Score Matching Case-control Study	Non-selected	4 years
Pollo-Flores, et al	2015	34	Randomized controlled trial	simvastatin	3 months
Huang, et al	2016	13,086	Propensity Score Matching Case-control Study	non-selected	12 years
Mohanty, et al	2016	1370	Propensity Score Matching Case-control Study	atorvastatin, fluvastatin, lovastatin, pravastatin, rosuvastatin and simvastatin	14 years
Simon, et al	2016	9135	Case-control Study	atorvastatin and fluvastatin	14 years
Abraldes, et al	2016	147	Randomized controlled trial	simvastatin	2 years
Bang, et al	2017	744	Case-control Study	non-selected	8 years
Chang, et al	2017	1350	Propensity Score Matching Case-control Study	non-selected	8.5 years
Kim, et al	2017	9852	Nested Case-control Study	atorvastatin, lovastatin, pravastatin, rosuvastatin and simvastatin	12 years
Wong, et al	2017	69,184	Propensity Score Matching Case-control Study	atorvastatin, fluvastatin, lovastatin, pravastatin, rosuvastatin and simvastatin	3 years
Wani, et al	2017	76	Self-control Study (Prospective Cohort)	simvastatin	3 months
Bishnu, et al	2018	23	Randomized controlled trial	atorvastatin	1 month
Kaplan, et al	2019	19,341	Propensity Score Matching Case-control Study	non-selected	5.5 years
Elwan, et al	2019	40	Randomized controlled trial	simvastatin	1 month

### Statin influenced parameters of hepatic portal vessel pressure in short-term treatment

**Fig. 2:**
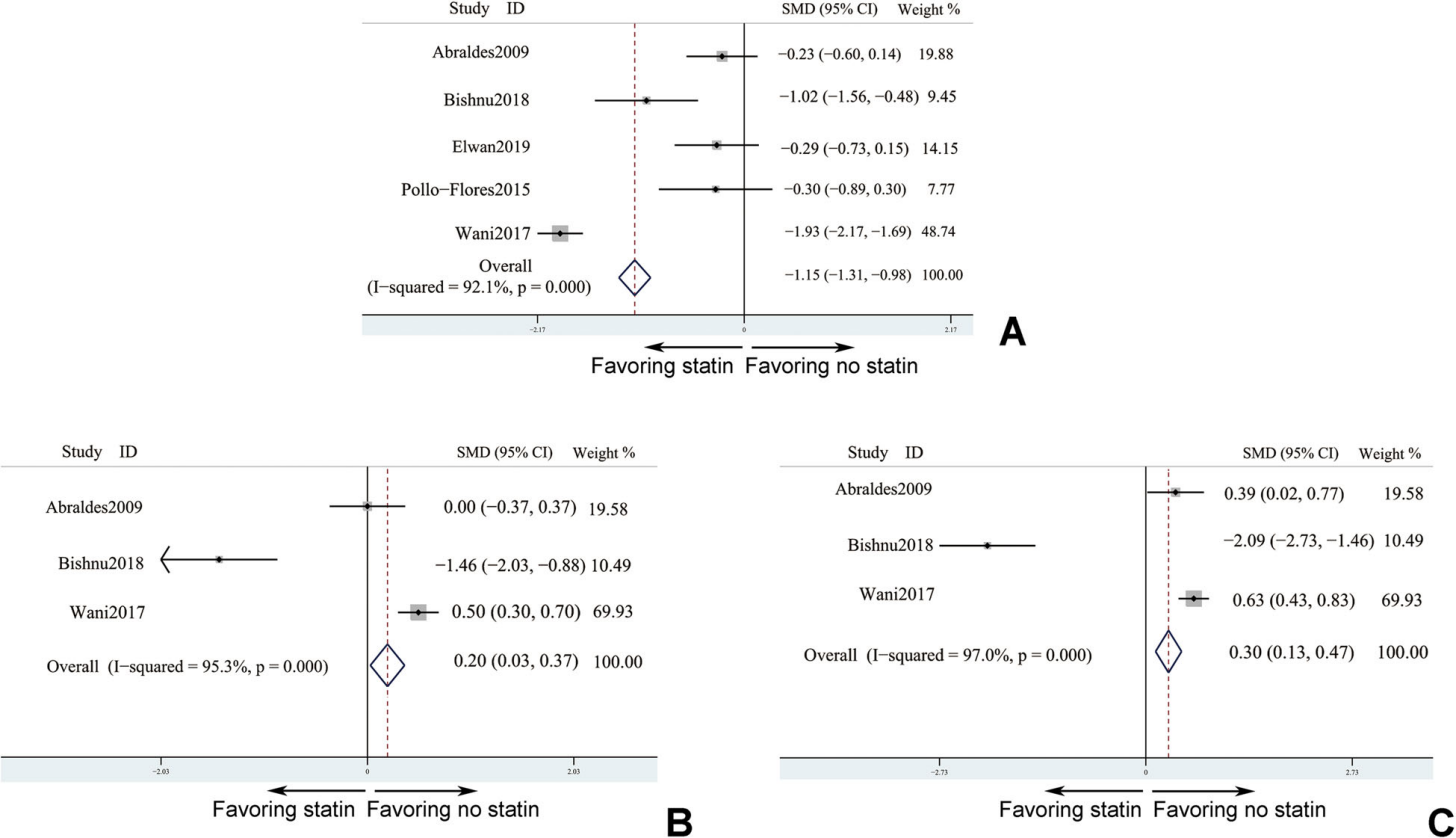
Statin influenced parameters of hepatic portal vessel pressure in short-term treatment. **a** hepatic venous pressure gradient (HVPG); **b** wedged hepatic venous pressures (WHVP); **c** free hepatic vein pressure (FHVP). 5 studies (**a**) and 3 studies (**b** & **c**) - Abraldes et al., [21]; Bishnu et al., [24]; Elwan et al., [26]; Pollo-Flores et al., [32]; and Wani et al., [35]

Five studies [21, 24, 26, 32, 35] reported parameters of hepatic portal vessel pressure with follow-up duration less than 3 months. In these studies, three important hepatic portal hemodynamic indexes, hepatic venous pressure gradient (HVPG), free hepatic vein pressure (FHVP), and wedged hepatic venous pressures (WHVP) which could reflect the degree of portal hypertension, were included. Since these parameters were continuous variables, pooled SMD was calculated. Statin treatment could significantly decrease the value HVPG comparing with control group (SMD_HVPG_ = − 1.146, 95% confidence interval (CI): − 1.3120-0.981, *I*^*2*^ > 50%). However, compared to patients without statin treating, patients receiving statin intervention was proved to fail to lower value of FHVP and WHVP (SMD_FHVP_ = 0.3, 95% CI: 0.13–0.47, *I*^*2*^ > 50% and SMD_WHVP_ = 0.2, 95% CI: 0.03–0.37, *I*^*2*^ < 50%), respectively. Consequently, HVPG was verified under condition of statin taking (Fig. 2a). Unfortunately, FHVP and WHVP may not be sensitive enough to detect the difference. It meant that even though a short-term exposure to statin, the portal hypertension could be alleviated (Fig. 2b & c).

### Prognosis of liver cirrhosis benefited from statin treatment in long term follow-up

**Fig. 3:**
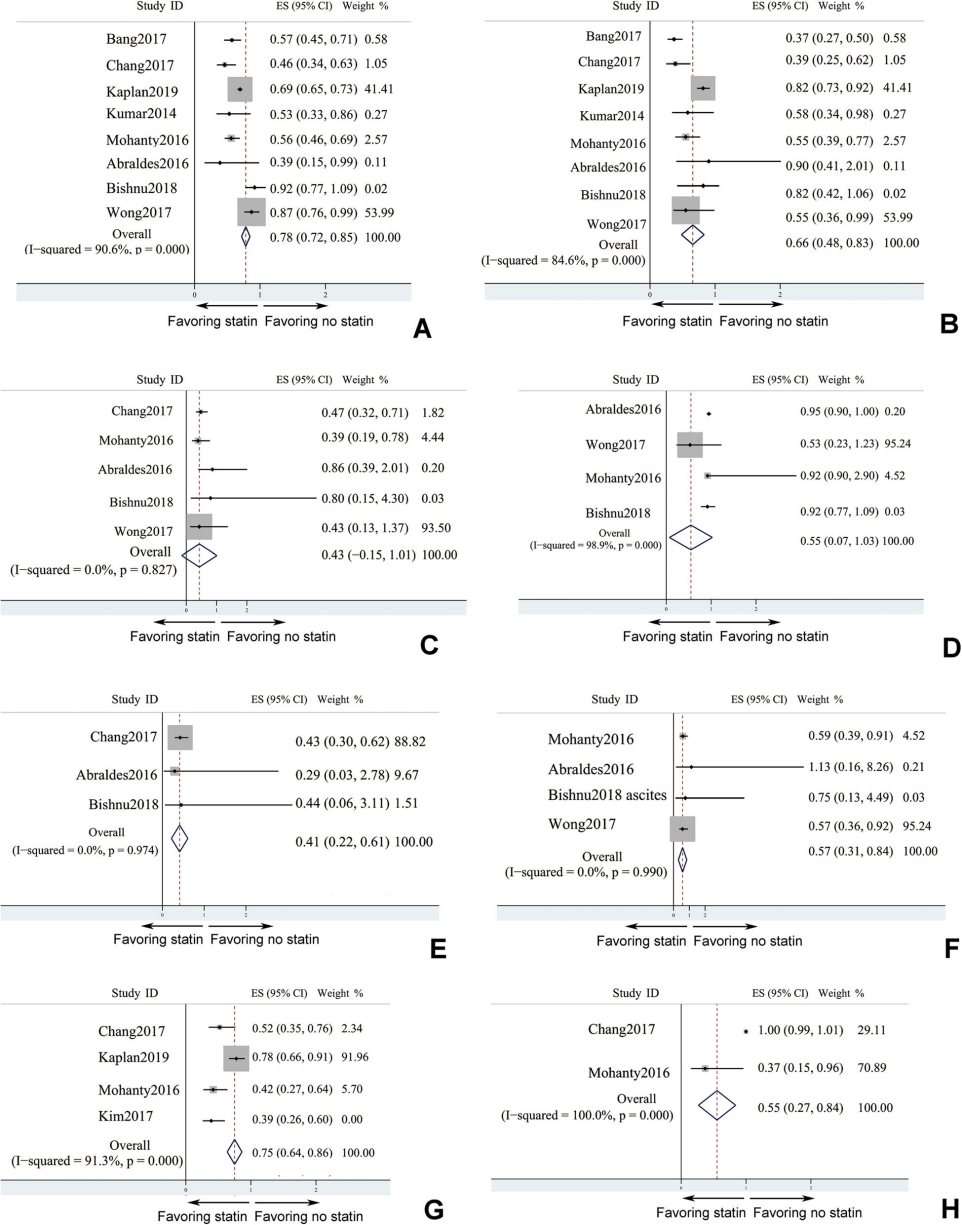
Prognosis of liver cirrhosis benefited from statin treatment in long term follow-up. **a** mortality; **b** decompensation events (8 studies - Bang et al., [23]; Chang et al., [25]; Kaplan et al., [28]; Kumar et al., [30]; Mohanty et al., [31]; Abraldes et al., [22]; Bishnu et al., [24]; and Wong et al., [36]); **c** esophageal variceal bleeding (5 studies); **d** spontaneous bacterial peritonitis (SBP) (4 studies); **e** hepatic encephalopathy (HE) (3 studies); **f** ascites (4 studies); **g** HCC (hepatocellular carcinoma) development (4 studies); **h** liver transplantation rate (2 studies)

Influence of statin in survival rate, decompensation events of liver cirrhosis, and HCC were investigated for long term follow-up as long as 14 years, and related data was extracted and analyzed to interpret the effect of statin. Statin treatment surprisingly improved survival rate in liver cirrhosis (HR = 0.782, 95% CI: 0.718–0.846, *I*^*2*^ > 50%), and the decreased risk of mortality as a hard clinical end-point persuasively verified the beneficial effects of statin (Fig. 3a). Decompensation of liver cirrhosis included variceal haemorrhage, hepatic encephalopathy, ascites, and even spontaneous bacterial peritonitis. Studies which had reported the incidence of the total decompensated cirrhosis events were analyzed, and the pooled data suggested statin treatment could decrease the occurrence of decompensated cirrhosis events (pooled HR = 0.658, 95% CI: 0.483–0.833, *I*^*2*^ < 50%) after long-term follow up. Although decompensation events of liver cirrhosis were decreased (Fig. 3b), subgroup analysis of each specific decompensated cirrhosis event was applied (Fig. 3c-f). Statin seemed not to decrease the risk of esophageal variceal bleeding (Fig. 3c) and spontaneous bacterial peritonitis (SBP) (Fig. 3d). However, statin was proved to decrease the risk of hepatic encephalopathy (Fig. 3f) and ascites (Fig. 3f). As one of the most serious complication, HCC could sharply increase the risk of mortality [45, 46]. Accordingly, effect of statin on lowering the occurrence of HCC (HR = 0.75, 95% CI: 0.64–0.86) should really alleviate the disease burden of liver cirrhosis (Fig. 3g, *I*^*2*^ > 50%). Logically, statin could decrease the case need for liver transplantation (Fig. 3h, *I*^*2*^ < 50%).

### Incidence of adverse events didn’t increase in statin users

**Fig. 4:**
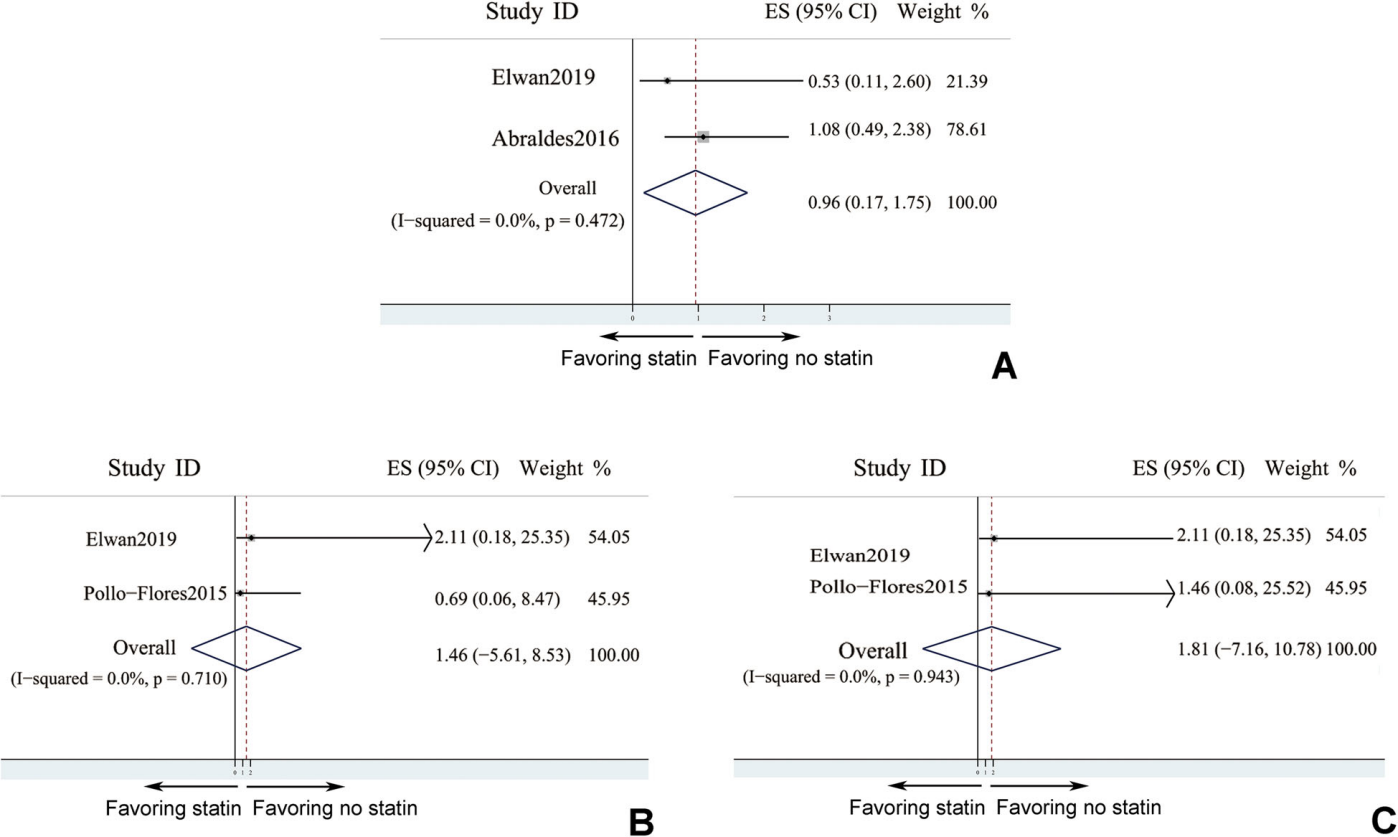
Incidence of adverse events didn’t increase in statin users. **a** worsened ascites; **b** myalgia; **c** diarrhea: 2 studies only

As is known to us that statin could lead to muscle injury and liver dysfunction, which might further result in myalgia and worsening of ascites. Therefore, the incidence of statin related adverse events was analysed here (Fig. 4). Based on existed studies, statin usage didn’t increase the number of cases of worsened ascites (pooled OR = 0.959, 95% CI: 0.169–1.749, *I*^*2*^ < 50%), in comparison with control group. Myalgia events were evenly distributed between different groups (pooled OR = 1.459, 95% CI: − 5.614 - 8.532, *I*^*2*^ < 50%), and the frequency of myalgia was comparable no matter stain was treated or not (Fig. 4b). Besides, statin was reported to correlate with gastro-intestinal problem, such as diarrhea. However, pooled data indicated that the number of diarrhea patients with statin treatment was not different from that in patients without statin treatment (pooled OR = 1.813, 95% CI: − 7.156 - 10.782, *I*^*2*^ < 50%). Therefore, statin might not increase risk of diarrhea in liver cirrhosis patients (Fig. 4c).

### Subgroup analysis by the study design of RCT versus non-RCT

Considering the concerns about any difference of statin effects between RCT study versus. Non-RCT study, therefore, subgroup analysis by the study design (whether RCT or not) was performed when the number of included literatures was enough for this purpose. End-point events involved in this part contained mortality, decompensation events, SBP, ascites, esophageal variceal bleeding. And it indicated the results of pooled data from RCT study was not consistent with results from non-RCT study, except for decompensation events and esophageal variceal bleeding (Additional file 2: Figure S2). Compared to RCT study, non-RCT study possessed with much more objects. And well-designed non-RCT study such as Propensity Score Matching Case-control Study.

Cohort study with much more amount of patients could also offer favorable evidence for clinical practice.

### Dose-dependent effects of statin on HCC development, decompensated cirrhosis events occurrence, and liver cirrhosis progression

**Fig. 5:**
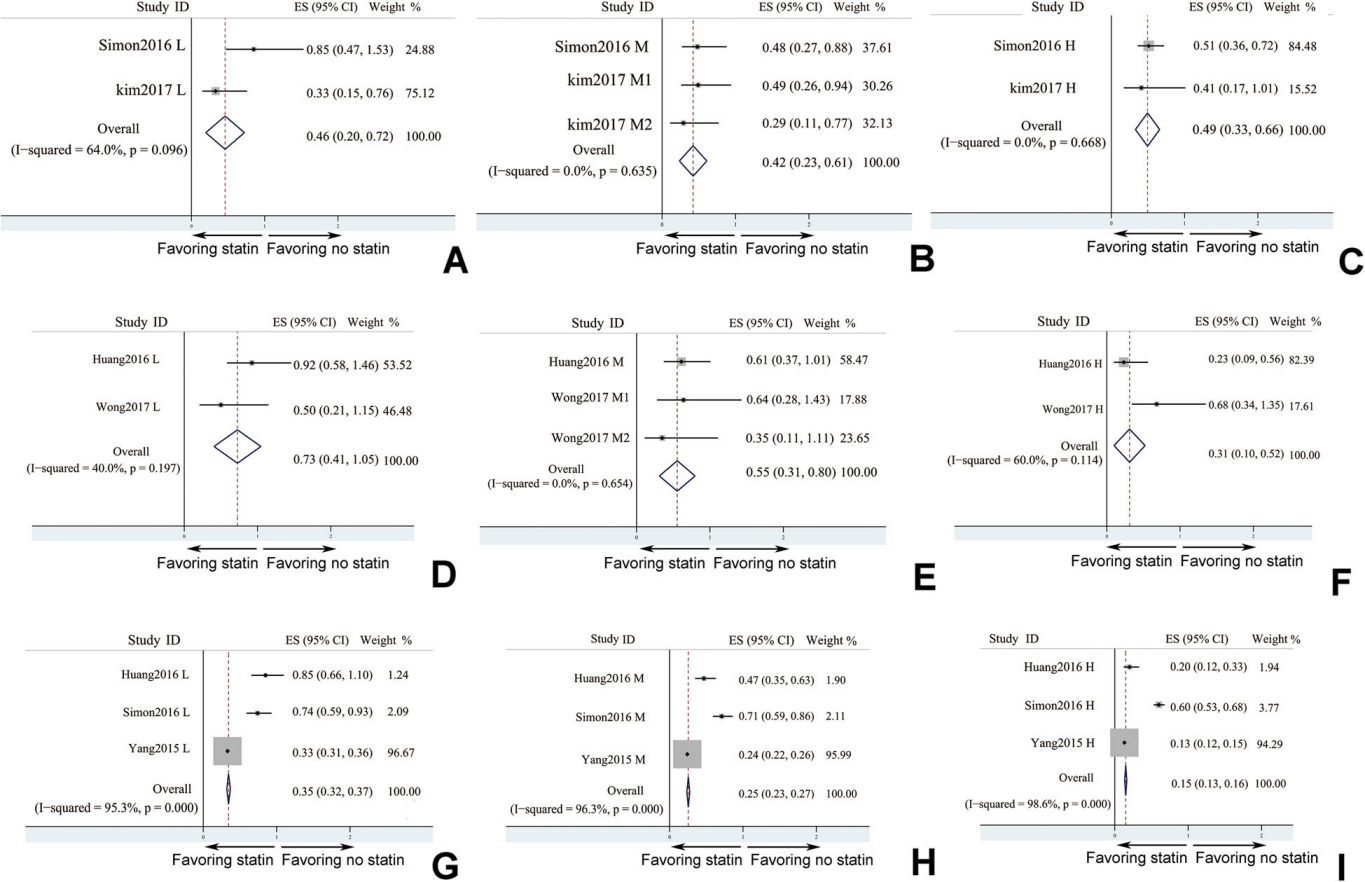
Dose-dependent effects of statin on HCC development, decompensation events occurrence, and liver cirrhosis progression. **a** HCC development; **b** decompensation events occurrence; **c** liver cirrhosis progression (L: low dose statin; M: medium dose statin; H: high dose statin): There are A to H figures and 2–3 studies. Please change as A-C: HCC development; **d**–**f** decompensation events occurrence; and **g**–**i** liver cirrhosis progression; L: low dose statin; M: medium dose statin; H: high dose statin

In each included studies, statin was divided into 3 doses: low dose, medium dose, and high dose. Effects of different dose of statin on liver cirrhosis were analyzed (Fig. 5). All 3 doses of statin could decrease of HCC (Fig. 5a) incidence (low dose: HR = 0.459, 95% CI: 0.195–0.724, *I*^*2*^ > 50%; medium dose: HR = 0.422, 95% CI: 0.235–0.609, *I*^*2*^ < 50%; high dose: HR = 0.494, 95% CI: 0.329–0.66, *I*^*2*^ < 50%). Low dose of statin didn’t influence decompensation of liver cirrhosis (HR = 0.726, 95% CI: 0.406–1.047, *I*^*2*^ < 50%). However, both medium dose and high dose of statin could decrease incidence of decompensation events of liver cirrhosis (medium dose: HR = 0.554, 95% CI: 0.311–0.798, *I*^*2*^ < 50%; high dose: HR = 0.31, 95% CI: 0.098–0.522, *I*^*2*^ > 50%). Liver puncture biopsy to evaluate liver cirrhosis pathological progression indicated that all doses of statin could mitigate liver fibrosis and sclerosis (low dose: HR = 0.345, 95% CI: 0.32–0.37, *I*^*2*^ > 50%; medium dose: HR = 0.254, 95% CI: 0.235–0.274, *I*^*2*^ > 50%; high dose: HR = 0.149, 95% CI: 0.135–0.164, *I*^*2*^ < 50%). It seemed that higher dose of statin tended to have better effect on relieving pathological progression of liver cirrhosis (Fig. 5c).

### Publication bias analysis

The representative publication bias analysis by Begg’s test showed a symmetrical distribution of included publications (*p* = 0.427) in a funnel plot (Additional file 1: Figure S1), and this indicated that there didn’t exist publication bias among articles included in present study.

## Discussion

In the present meta-analysis, 17 studies were finally included for data pooling and synthesis. Statin was proved to be effectively lowering the risk of the occurrence of decompensated liver cirrhosis such as variceal haemorrhage, encephalopathy, and spontaneous bacterial peritonitis, which was treated as life-threatening event in chronic liver cirrhosis in a long-term follow-up. Besides, statin could decrease the incidence of HCC which was a serious complication of liver cirrhosis. In addition, the dose-dependent effect of statin in liver cirrhosis was testified base on pooled data, and it indicated that statin had potential in treating chronic liver disease. Even in short-term therapeutic of statin, the hemodynamics of portal vessel was significantly improved. Since it drew concerning about the statin application in chronic liver cirrhosis might accentuate liver dysfunction, we compared the drugs related adverse events between statin treated group and non-statin treated group. Diarrhea, myalgia, and ascites accentuation showed no difference no matter statin was used or not. This study was characterized with the largest sample size to comprehensively evaluate the efficacy and safety of statin on liver cirrhosis and its development. In spite of results mentioned above, for fear of any difference of statin effects between RCT study versus. Non-RCT study, subgroup analysis by the study design (whether RCT or not) was performed. It indicated the results of pooled data from RCT study was not consistent with results from non-RCT study, except for decompensation events and esophageal variceal bleeding. To our knowledge, similar systematic study was hardly seen before.

As one of the mostly prescribed medication, statin is widely used in the primary prevention of coronary ischemic heart disease by outstandingly inhibiting the activity of HMG-CoA. However, laboratory studies showed that statin could further attain endothelial functional improvement independently from down-of cholesterol level [20, 47]. Previous investigation indicated that statin could improve the resilience and compliance of portal vessels by promoting the production of vascular endothelium-derived relaxing factor, namely, nitric oxide [48–52]. Furthermore, clinical studies hinted that statin could mitigate hepatic portal hypertension as well with a short therapeutic duration (mean value of follow-up period: 3 months) [21, 24, 26, 32, 35]. Moreover, statin was proved to function as a kind of free radical eliminated agent which could relieve oxidative stress reaction in liver cirrhosis progression [53, 54]. Inflammatory reaction could be suppressed by statin through inhibiting and eliminating the over-produced free radical or other pernicious by-product in liver cirrhosis [55, 56], and hence hepatic cell injury and fibrosis could be partly prevented from underlying this mechanism. Given myalgia (muscular damage and creatine kinase elevation) as one of the most common drug-related adverse reactions clinical studies were designed to assess its incidence in statin treated liver cirrhosis, and most of which confirmed the safety of statin use [26, 32]. Portal hypertension as a marker of decompensated liver cirrhosis could further exacerbate liver cirrhosis to form a vicious cycle [57–59], and statin could break this circle by lowering hepatic portal vascular pressure to improve the prognosis of liver cirrhosis. HCC could be evolved from sustained condition of liver cirrhosis [60], and statin might decrease the occurrence rate of HCC through slowing the development of disease course of liver cirrhosis. Studies ranging from bench to bed indicated that chronic liver cirrhosis might be a novel indication for statin treatment, and pooled data of clinical studies finally supported this viewpoint. In cardiovascular disorders, especially coronary atherosclerosis disease (CAD), statin treatment showed eminent dose-dependent effects on the prognosis of CAD [61, 62]. Similarly, statin also exhibited dose-dependent effects on HCC development, decompensated cirrhosis events occurrence, and liver cirrhosis progression. Despite low dose of statin didn’t affect decompensated liver cirrhosis, both medium and high dose of statin could improve decompensated liver cirrhosis. Furthermore, higher dose of statin tended to have better effect on relieving pathological progression of liver cirrhosis.

A systematic review has already been done to quantitatively summarize effects of statin and accentuate the important role of statin in treating chronic liver disease. Based on this study, statin use is probably associated with lower risk of hepatic decompensation and mortality, and might reduce portal hypertension, in patients with chronic liver diseases [13]. Nonetheless, this study failed to evaluate the safety of statin, and the number of studies it included was less than ours. To our knowledge, similar systematic study with multi-dimension and statistical depth was hardly seen before. The quality of the present meta-analysis was guaranteed by thorough retrieval strategy, well-defined inclusion and exclusion criteria, guideline mediated literature evaluation, and strictly quantitative analysis by well-acknowledged STATA software.

### Limitation

This study included 17 studies, parts of which were of characterized with observational and case-control design. The included articles had defects such as no randomization, retrospective design, and small scale, and these flaws could somehow devaluate the quality of our study. However, studies with high quality were involved with high weighting ratio, which meant that study with higher quality contributed more on the present meta-analysis. The included studies investigated liver disease with different aetiology, such as alcoholic liver disease, NAFLD, HBV, HCV, and so on. As a result, the heterogeneity of liver cirrhosis aetiology at baseline might lead to bias of treatment response to statin. In addition, the present regarded different kinds of statin, such as simvastatin, artovastatin, fluvastatin, and so on, as a whole, however, the head-to-head comparison of effects of different kinds of statin on liver cirrhosis should be discussed in future. Perhaps, a network meta-analysis could solve this problem. Furthermore, the limited number of articles eligible for different research target made sensitivity analysis not applicable. Additionally, patients with β-blocker administration or comorbidities of chronic kidney disease were also susceptible to exacerbated hepatic function, and these confounding factors were not presented in the included studies. Therefore, risk-stratified analysis couldn’t be carried out. Consequently, large scale, prospective, multi-center, and randomized clinical trials are still highly needed with clearly reported confounding factors.

## Conclusion

In the present study, statin was proved to be effectively lowering the risk of the occurrence of decompensated liver cirrhosis such as encephalopathy and ascites, which was treated as life-threatening event in chronic liver cirrhosis in a long-term follow-up. Unfortunately, statin might have no effect on variceal haemorrhage and spontaneous bacterial peritonitis. Besides, statin could decrease the incidence of HCC which was a serious complication of liver cirrhosis. In addition, the dose-dependent effect of statin in liver cirrhosis was testified base on pooled data, and it indicated that higher dose of statin tended to have better effect on relieving pathological progression of liver cirrhosis. Even in short-term therapeutic of statin, the hemodynamics of portal vessel was significantly improved. Drugs related adverse events between statin treated group and non-statin treated group show no difference. This study was characterized with the largest sample size to comprehensively evaluate the efficacy and safety of statin on liver cirrhosis and its development.

## Supplementary information

**Additional file 1: Figure S1.** Typical diagram of publication bias analysis.

**Additional file 2: Figure S2.** Subgroup analysis by the study design of RCT versus non-RCT. A: mortality in RCT study; B: mortality in non-RCT study; C: decompensation events in RCT study; D: decompensation events in non-RCT study; E: spontaneous bacterial peritonitis (SBP) in RCT study; F: spontaneous bacterial peritonitis (SBP) in non-RCT study; G: ascites in RCT study; H: ascites in non-RCT study; I: esophageal variceal bleeding in RCT study; J: esophageal variceal bleeding in non-RCT study.

## Data Availability

The datasets used and/or analysed during the current study are available from the corresponding author on reasonable request.

## Abbreviations

CAD: Coronary Atherosclerosis Disease
CI: Confidence interval
FHVP: Free Hepatic Vein Pressure
HCC: Hepatocellular carcinoma
HMG-CoA: 3-hydroxy-3-methylglutaryl co-enzyme A
HR: Hazard Ratio
HVPG: Hepatic Venous Pressure Gradient
OR: Odds Ratio
PRISMA: Preferred Reporting Items for Systematic Reviews and Meta-Analyses
SBP: Spontaneous bacterial peritonitis
SMD: Standard Mean Difference
WHVP: Wedged Hepatic Venous Pressures
