# Supplementary figures and images for: Comprehensive evaluation of effects and safety of statin on the progression of liver cirrhosis: a systematic review and meta-analysis
Source: BMC Gastroenterol. 2019 Dec 30;19:231. doi: 10.1186/s12876-019-1147-1 (PMC6938024; doi:10.1186/s12876-019-1147-1)

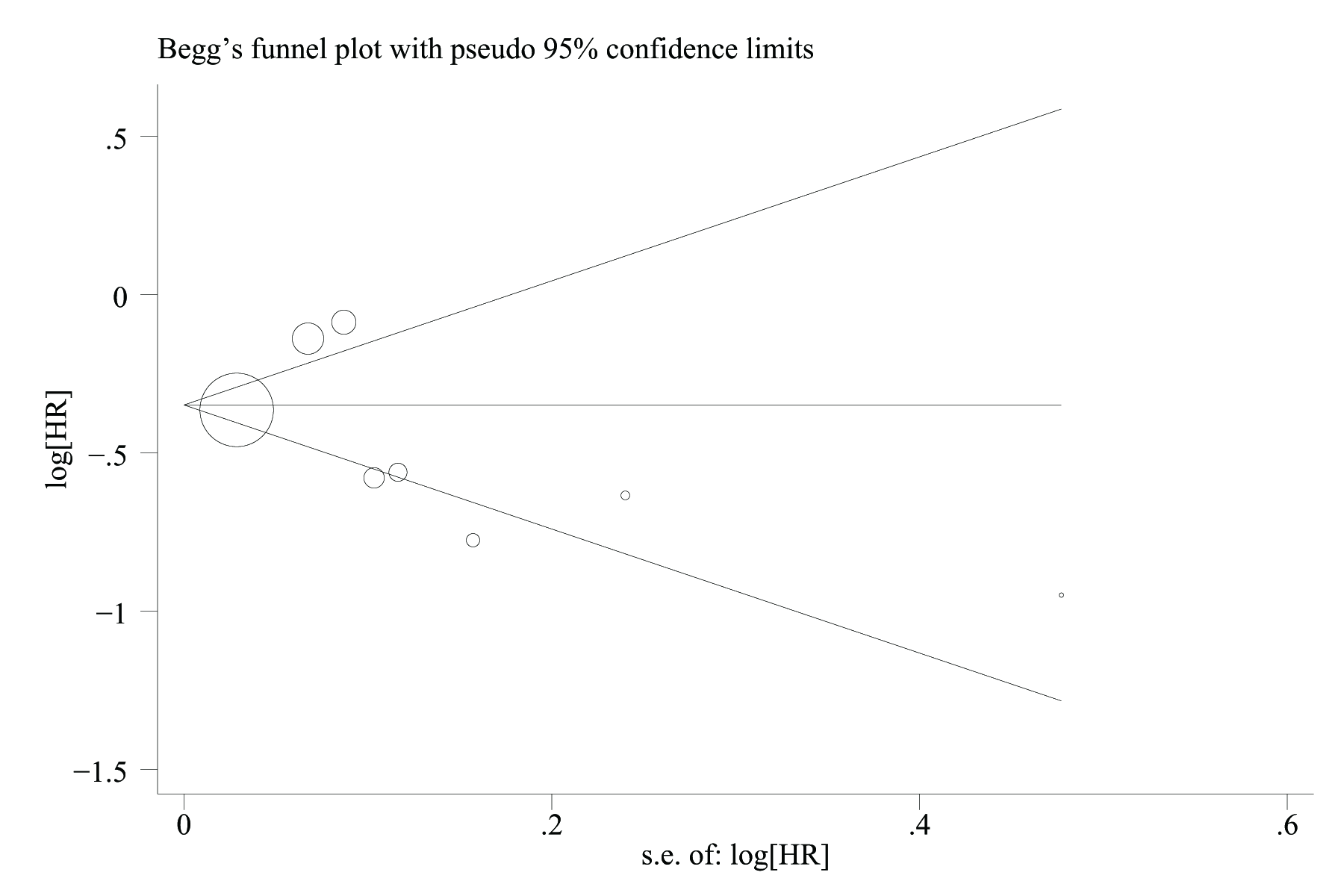

Supplement: Supplementary file 1 — Additional file 1: Figure S1. Typical diagram of publication bias analysis. [file 12876_2019_1147_MOESM1_ESM.tif]

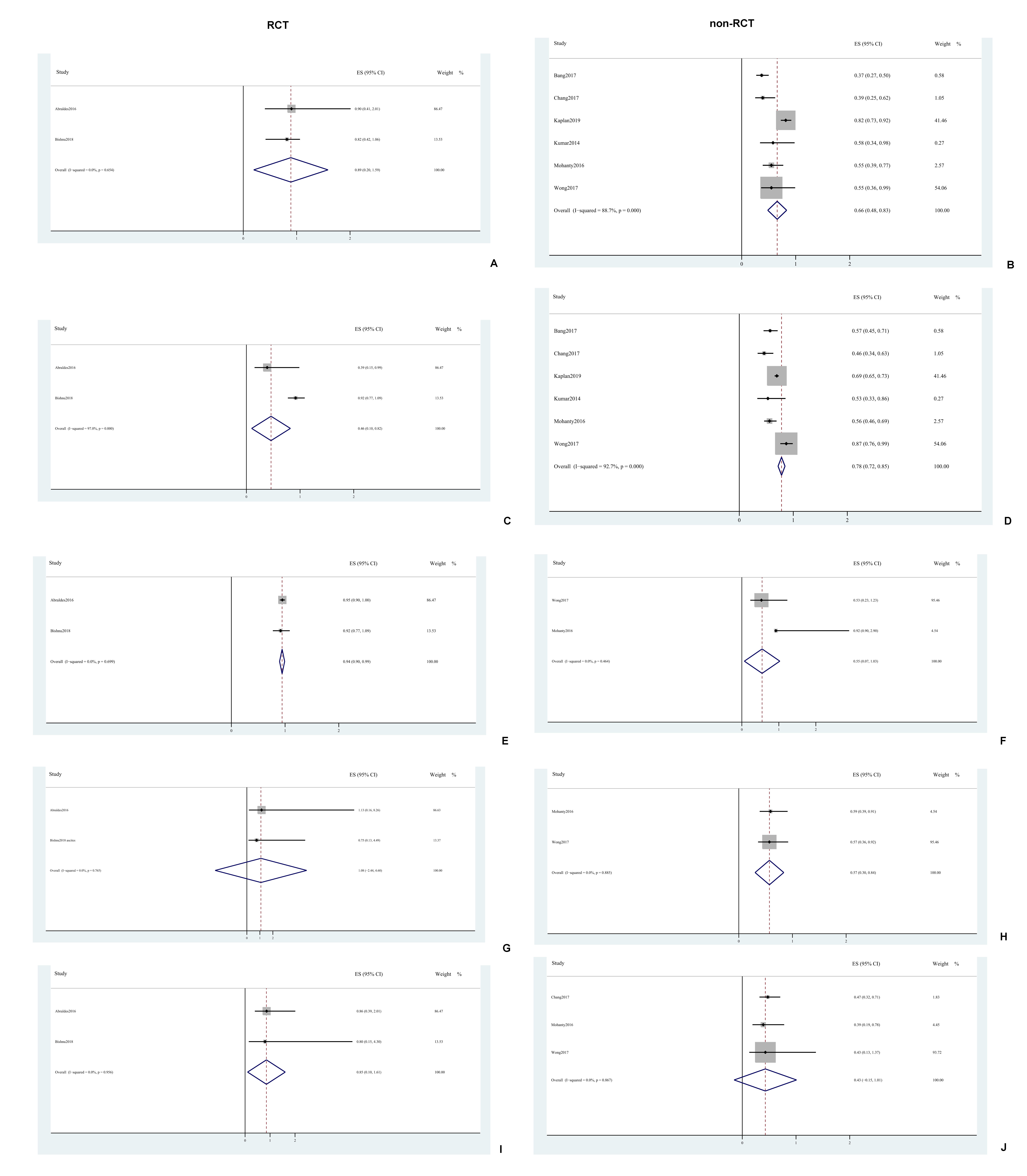

Supplement: Supplementary file 2 — Additional file 2: Figure S2. Subgroup analysis by the study design of RCT versus non-RCT. A: mortality in RCT study; B: mortality in non-RCT study; C: decompensation events in RCT study; D: decompensation events in non-RCT study; E: spontaneous bacterial peritonitis (SBP) in RCT study; F: spontaneous bacterial peritonitis (SBP) in non-RCT study; G: ascites in RCT study; H: ascites in non-RCT study; I: esophageal variceal bleeding in RCT study; J: esophageal variceal bleeding in non-RCT study. [file 12876_2019_1147_MOESM2_ESM.tif]
